# lncRNA HOTAIR functions and therapeutic perspectives

**DOI:** 10.18632/oncoscience.563

**Published:** 2022-09-13

**Authors:** Sabrina Garbo, Marco Tripodi, Cecilia Battistelli

**Affiliations:** ^1^Department of Molecular Medicine, Sapienza University of Rome, Rome 00161, Italy; ^2^National Institute for Infectious Diseases “L. Spallanzani”, Rome 00149, Italy

**Keywords:** HOTAIR, lncRNA, EMT, cancer, RNA-based therapy

## Abstract

Long non-coding RNAs (lncRNAs) exert central pathophysiological roles through the regulation of gene expression both at transcriptional and post-transcriptional levels. The characterization of lncRNAs’ interactome is disclosing several new mechanisms that control disease onset and progression thus opening the way to the development of new pioneering therapeutic approaches. Regarding the lncRNA HOTAIR, found upregulated in several cancers and in liver fibrosis, it has been proved as a potential therapeutic target. HOTAIR acts as a ceRNA for several miRNAs and it directly interacts with chromatin remodelling complexes (e.g. PRC2 and LSD1/NuRD complexes). In this regard, we recently reported the transcription factor SNAIL-mediated recruitment of HOTAIR/PRC2 complex on specific chromatin sites causing epithelial genes’ repression through epigenetic chromatin modifications. Conversely, HOTAIR is repressed by the liver-enriched transcriptional factor HNF4a that binds to both HOTAIR promoter and distant enhancer and impairs the formation of a chromatin loop between these genomic regions.

In a therapeutic perspective, we design and validated the first example of a dominant negative lncRNA molecule (HOTAIR-sbid) that covers the HOTAIR portion involved in the interaction with SNAIL while is devoid of the domain of interaction with EZH2. Functionally, HOTAIR-sbid expression impairs SNAIL/EZH2/endogenous HOTAIR interaction; thus, PRC2 complex is not recruited on SNAIL-target chromatin sites (i.e. epithelial genes’ promoters). Accordingly, the cells rescue an epithelial phenotype, reduce EMT and, in turn, migratory, invasive and anchorage independent growth abilities. This approach promises high level of specificity and limited off-target effects. Future investigations should enhance RNAs’ stability and should design strategies for the delivery of these molecules to specific target cells.

Long non-coding RNAs (lncRNAs), an important class of RNAs more than 200 nucleotides long, that acquired attention only in the last decades due to their subtle phenotype, are now recognized to exert central pathophysiological roles. The efforts in highlighting their functions, structures and interactors (other RNAs, DNA and proteins) and in the development of experimental approaches to detect these interactomes, revealed their pivotal role in regulating gene expression both at transcriptional and post transcriptional level. In particular this apply to lncRNAs involved in several tumours’ onset and dissemination where a remarking contribution of basic and applied research now opens the way to the development of new pioneering therapeutic approaches.

The lncRNA Hox Antisense Intergenic RNA (HOTAIR) represents one of the most studied and characterized lncRNAs; it is encoded within the Homeobox C (HOXC) locus and is found upregulated in several cancers [1, 2], in CCl4-induced mouse liver fibrosis models, human fibrotic livers and hepatic stellate cells (HSCs) activated upon TGF-β1 stimulation [3]. Therefore, HOTAIR has been recognized as a potential therapeutic target in future clinical management. Molecularly, it has been found that HOTAIR acts as a ceRNA for several miRNAs like miR-148b regulating DNMT1/MEG3/p53 pathways in HSCs [3], miR-217-5p influencing p-PI3K/p-AKT/MMP-2/9 protein expression [4], miR-206 repressing CCL2 [5], miR-93 targeting ATG12 and influencing radiosensitivity [6], miR-526b-3p downregulating DHX33 and apoptosis [7], miR-214-3p repressing FLOT1 expression and tumour growth [8].

In parallel, HOTAIR was found to directly interact with chromatin remodelling complexes like Polycomb repressive complex 2 (PRC2) and the LSD1/NuRD complex [9]. In this regard, we recently reported that HOTAIR directly interacts with the catalytic subunit of the PRC2 complex, EZH2, and with the sequence-specific transcription factor SNAIL, driving the transcriptional repression of SNAIL-target genes during the epithelial to mesenchymal transition [9]. Mechanistically, SNAIL recognizes specific chromatin sites on the promoter regions of its target genes after the induction of EMT and requires to interact with HOTAIR to exert its repressive activity. In fact, HOTAIR recruits EZH2 thus inducing the trimethylation of H3K27 on the regulatory regions of epithelial genes like E-cadherin and HNF1/4.

Conversely, the liver-enriched transcriptional factor HNF4a, in order to drive hepatocyte differentiation and promote mesenchymal to epithelial transition (MET), negatively regulates HOTAIR expression through its binding to both HOTAIR promoter and distant enhancer. This in turn impairs the formation of a chromatin loop between these genomic regions that promotes HOTAIR transcription [10].

In a therapeutic perspective, accumulating evidence revealed that ncRNAs are potential druggable targets, due to their functions and structure. In this frame, the majority of the reports evaluated the effect of lncRNA knockdown in preventing disease onset and progression, including cancer. The most recognized and shared approach is based on the use of shRNAs that induce a downregulation of lncRNA expression by RNA interference. This strategy allows a general silencing of the lncRNA but does not selectively target specific functions. Differently, we recently reported the first example of a dominant negative RNA molecule that competes with the endogenous HOTAIR lncRNA in a specific function. This molecule, named HOTAIR-sbid (for Snail-binding domain), covers the HOTAIR portion involved in the interaction with SNAIL while is devoid of the domain of interaction with EZH2. Molecularly, HOTAIR-sbid was shown to impair SNAIL/EZH2 interaction and the SNAIL binding to the endogenous HOTAIR. Therefore, PRC2 complex is not recruited on SNAIL/HOTAIR target sites and SNAIL/HOTAIR-dependent epigenetic transcriptional repression of epithelial genes (E-cadherin, HNF1a, HNF4a) is impeded. Functionally, this dominant negative-based strategy, reflects on the rescue of an epithelial phenotype, with reduction of migratory, invasive and anchorage-independent growth in HCC cells and notably prevents TGFβ-induced EMT in hepatocytes [11]. These results pave the way for future investigations aimed to determine the level of specificity and/or the off- targets effects of an HOTAIR competing approach. The understanding of the site of specific interaction among specific lncRNAs and specific proteins may encourage the setup of delivery strategies that protect these RNAs from degradation and vehicular these molecules to specific target cells.
